# AcrVA3 Is a Double Strand DNA‐Cleaving Anti‐CRISPR That Indirectly Inhibits Cas12

**DOI:** 10.1096/fj.202502278RR

**Published:** 2026-03-19

**Authors:** Ju Hee Han, Young Jun Kang, So Yeon Lee, Hyo Been Jin, Chang Sup Lee, Hyun Ho Park

**Affiliations:** ^1^ College of Pharmacy Chung‐Ang University Seoul Republic of Korea; ^2^ Department of Global Innovative Drugs, Graduate School of Chung‐Ang University Seoul Republic of Korea; ^3^ College of Pharmacy and Research Institute of Pharmaceutical Science Gyeongsang National University Jinju Republic of Korea

## Abstract

CRISPR‐Cas12 systems protect bacteria from foreign DNA, but are themselves targeted by anti‐CRISPR (Acr) proteins evolved by phages. Among the eight known AcrV proteins that inhibit Cas12‐based type V CRISPR‐Cas systems, the mechanisms of all except AcrVA3 have been structurally and biochemically characterized. Here, we report the high‐resolution structure of AcrVA3 and examine its inhibitory function in vitro. Unexpectedly, AcrVA3 does not directly work on Cas12. Instead, it exhibits double‐stranded DNA (dsDNA) cleavage activity, suggesting an indirect mechanism of CRISPR inhibition through DNA degradation. This unique DNA‐centric strategy contrasts with previously known Acr mechanisms and expands our understanding of how mobile genetic elements evade CRISPR immunity.

## Graphical abstract

This study reports the high‐resolution structure of the anti‐CRISPR protein AcrVA3 and reveals its unexpected inhibitory mechanism against Cas12. AcrVA3 does not directly interact with Cas12 but instead exhibits double‐stranded DNA cleavage activity. By degrading the DNA substrate required for CRISPR interference, AcrVA3 indirectly suppresses Cas12 function, uncovering a previously unrecognized anti‐CRISPR strategy.

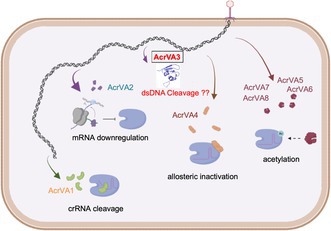

## 1 Introduction

CRISPR–This work was supported by the National Research Foundation of Korea immunity in bacteria and archaea by recognizing and cleaving invading nucleic acids such as plasmids and phages [1]. Among these, the type V CRISPR‐Cas system, represented by Cas12 nucleases, has gained particular attention not only for its natural diversity but also for its biotechnological applications, including genome editing and diagnostics [2, 3]. However, as CRISPR‐Cas systems impose selective pressure on mobile genetic elements (MGEs), many phages have evolved counter‐defense strategies, including the expression of anti‐CRISPR (Acr) proteins that inhibit CRISPR‐mediated immunity [4]. These Acr proteins represent a rapidly expanding and mechanistically diverse family of natural CRISPR inhibitors [5].

**FIGURE 1:**
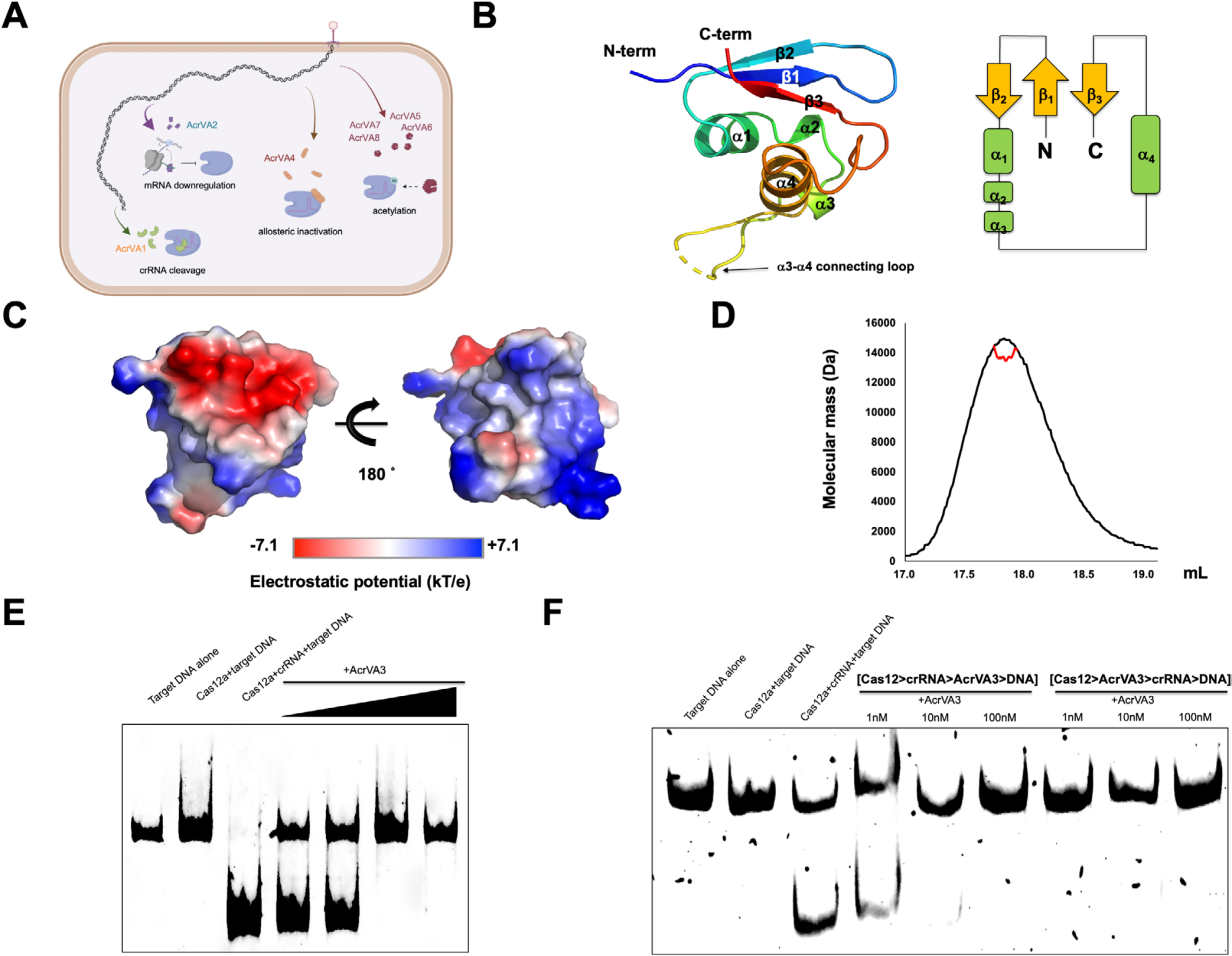
The overall structure and confirmation of anti‐CRISPR capability of AcrVA3. (A) A schematic diagram illustrating the known mechanisms of AcrVA family. (B) Ribbon and topology diagrams of the AcrVA3 structure. (C) Electrostatic surface representation of AcrVA3. (D) MALS profile from the SEC peak. (E) Anti‐CRISPR assay using AcrVA3 and Cas12a. (F) Order‐dependent Acr activity of AcrVA3.

To date, approximately 100 Acr proteins have been identified and only eight AcrVA proteins (AcrVA1 to AcrVA8) have been reported to target type V CRISPR‐Cas systems [6, 7]. Among the eight known AcrVA proteins, structural and biochemical studies have elucidated the inhibitory mechanisms of all except AcrVA3. AcrVA proteins inhibit Cas12 through crRNA cleavage (AcrVA1) [8], disruption of Cas12 biogenesis (AcrVA2) [7], allosteric binding (AcrVA4) [8], or lysine acetylation (AcrVA5–8) [9] (Figure 1A). While phage spotting assays have confirmed that AcrVA3 possesses anti‐CRISPR activity [2], its precise mechanism of Cas12 inhibition has remained completely unknown.

In this study, we determined the high‐resolution structure of AcrVA3 and demonstrated its ability to inhibit Cas12 activity in an in vitro system. Interestingly, we found that AcrVA3 does not directly bind to Cas12. Instead, it exhibits the unexpected ability to cleave double‐stranded DNA (dsDNA), suggesting an indirect mechanism of CRISPR‐Cas inhibition.

Our findings not only resolve the long‐standing question of how AcrVA3 inhibits Cas12, but also highlight a previously uncharacterized strategy employed by Acrs to evade CRISPR interference. This DNA‐centric mechanism expands our understanding of anti‐CRISPR diversity and suggests new possibilities for modulating CRISPR activity through indirect nucleic acid targeting.

## 2 Results and Discussion

To understand the function of AcrVA3, we determined its structure using recombinant protein. SEC revealed three peaks containing AcrVA3 (Figure S1), but only the second peak yielded crystals suitable for structure determination. We determined the structure at a high resolution of 1.73 Å, and the final refined model showed R_work_ and R_free_ values of 21.7% and 24.5%, respectively.

The AcrVA3 structure consists of four α‐helices and a mixed β‐sheet in a compact fold (Figure 1B). This unique architecture is distinct from known Acr proteins. *B*‐factor analysis shows the structure is largely rigid, with localized flexibility in the α3–α4 loop region. Electrostatic surface analysis of AcrVA3 revealed a strongly negative potential near the β‐strands, while the α3–α4 region showed a highly basic surface enriched in positive charges (Figure 1C).

Many anti‐CRISPR proteins are known to function as dimers or higher‐order oligomers [10, 11]. To determine the oligomeric state of AcrVA3, we performed multi‐angle light scattering (MALS) analysis. The results showed that AcrVA3 exists as a monomer in solution, with a calculated molecular weight of 13.8 kDa, which is in good agreement with the predicted monomeric mass of 12.6 kDa (Figure 1D). This indicates that AcrVA3 exists as a monomer in solution, and it is predicted to exert its function in a monomeric form.

Since there has been no direct evidence demonstrating that AcrVA3 inhibits Cas12 activity, we performed an in vitro Cas12 inhibition assay. The results showed that AcrVA3 suppressed Cas12 activity in a dose‐dependent manner (Figure 1E), providing the first direct biochemical evidence of its inhibitory function.

It is known that the inhibitory effect of Acr on Cas depends significantly on the order in which the components are added in the assay [12]. Therefore, we tested two different reaction sequences: first, pre‐incubating Cas12 with crRNA followed by addition of Acr and then target DNA; and second, pre‐incubating Cas12 with Acr before adding crRNA and target DNA. Our results showed that AcrVA3 effectively inhibited Cas12 activity in both setups, with a stronger inhibition observed when AcrVA3 was added before Cas12 encountered crRNA (Figure 1F).

To infer the functional mechanism of AcrVA3, we identified structurally similar proteins using the DALI server [13]. This analysis identified structural homologs with low similarity, suggesting AcrVA3 represents a novel fold (Figure S2). The protein most structurally similar to AcrVA3 was identified as the endonuclease I‐HmuI [14], and among known Acr proteins, AcrIIA15 showed the highest structural similarity [15]. I‐HmuI is an endonuclease that cleaves DNA [14]. AcrVA3 superimposes well with I‐HmuI, with an RMSD of approximately 1.9 Å, indicating a high degree of structural similarity. However, the sequence identity between the two proteins is very low, at around 15%.

**FIGURE 2:**
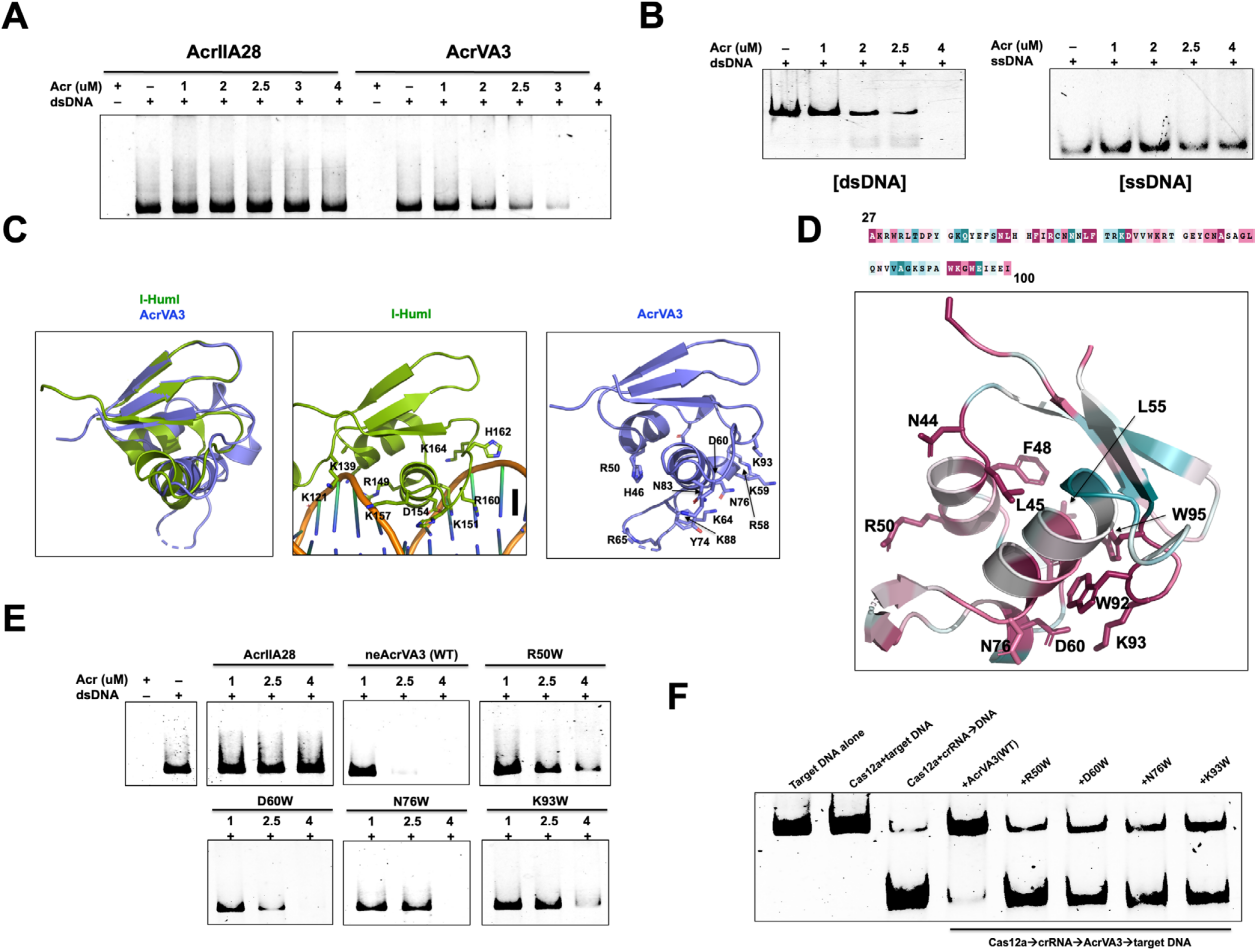
AcrVA3 inhibits the CRISPR‐Cas system by possessing dsDNA cleavage activity. (A) In vitro dsDNA binding or cleavage assay of AcrVA3. (B) In vitro dsDNA and ssDNA cleavage assay. (C) Structural comparison of AcrVA3 with structural homologue, I‐Hmul. The first panel is the superposed cartoon figure. Structural detail of dsDNA binding region of I‐Hmul and the region of AcrVA3 corresponding to the dsDNA‐binding site of I‐HmuI are provided at the second and third panel, respectively. (D) The result of ConSurf. Completely conserved residues are labeled. (E) dsDNA cleavage assay with wild‐type AcrVA3 and its various mutants. (F) Anti‐CRISPR assay with wild‐type AcrVA3 and its various mutants.

Notably, I‐HmuI and AcrIIA15 are known to function in DNA binding [14, 15]. Therefore, based on these structural similarities, we hypothesized that AcrVA3 may also be involved in DNA‐related functions. To test this hypothesis, we incubated AcrVA3 with DNA and observed that at concentrations above 2 μM, AcrVA3 induced double‐strand DNA (dsDNA) degradation in a dose‐dependent manner (Figure 2A). In contrast, AcrIIA28—a protein of similar size that acts on Cas9 rather than DNA [16]—did not cause any detectable changes in the DNA (Figure 2A). The presence of DNA fragments in the AcrVA3 reaction further confirmed that AcrVA3 induces DNA degradation (Figure 2B). Interestingly, this activity was not observed with single‐stranded DNA (ssDNA) (Figure 2B), suggesting that AcrVA3 specifically acts on dsDNA. To assess whether AcrVA3's DNA cleavage depends on specific sequences or structures, we treated plasmid DNA containing both supercoiled and linear forms. AcrVA3 cleaved both efficiently, with clearer fragmentation in larger plasmids, indicating that it targets dsDNA broadly, rather than in a sequence‐ or structure‐specific manner (Figure S3).

To gain insight into how AcrVA3 acts on dsDNA, we compared the structural details of AcrVA3 with the DNA‐bound structure of I‐HmuI (Figure 2C) [14]. In the case of I‐HmuI, DNA binding is known to involve a basic patch composed of K121, K139, R149, K151, K157, and R160, with a central acidic residue, D154, playing a critical role (Figure 2C) [14]. Similarly, in AcrVA3, we observed a corresponding basic patch formed by R50, R58, K59, K64, K88, and K93, with a central acidic residue, D60, positioned analogously (Figure 2C). In addition, we used ConSurf [17] to analyze functionally important, evolutionarily conserved residues. This analysis revealed that residues such as N44, L45, R50, L55, R55, D60, N76, W92, K93, and W95 are highly conserved (Figure 2D). Based on the structural comparison and conservation analysis, we predicted that R50, D60, N76, and K93 are likely involved in DNA binding. To test their involvement, we individually mutated each of these residues to tryptophan. As expected, the DNA cleavage activity of these mutants was significantly reduced compared to the wild‐type protein (Figure 2E). This result supports the conclusion that these residues play a critical role in DNA binding and cleavage by AcrVA3.

Finally, to determine whether the DNA‐cleavage‐defective AcrVA3 mutants retain the ability to inhibit Cas12, we performed a Cas12 inhibition assay using these mutants. The results showed that mutants with reduced DNA cleavage activity also exhibited a marked decrease in their ability to inhibit Cas12 compared to the wild‐type protein (Figure 2F). Based on these findings, we conclude that AcrVA3 is a DNA‐cleaving anti‐CRISPR protein, and its DNA cleavage activity may be essential for effective inhibition of Cas12. This mechanism suggests that AcrVA3 functions by masking or modifying the substrate rather than the nuclease itself, providing a novel paradigm of anti‐CRISPR activity.

## Author Contributions

H.H.P. designed and supervised the project. J.H.H. performed all the experiments, including collecting biochemical and structural data. J.H.H. and S.Y.L. solved the structure. Y.J.K. and H.B.J. performed MALS. H.H.P., J.H.H., and Y.J.K. wrote the manuscript. All the authors discussed the results and commented on the manuscript.

## Funding

This work was supported by the National Research Foundation of Korea (NRF) (RS‐2025‐02316334 and RS‐2026‐25470081).

## Conflicts of Interest

The authors declare no conflicts of interest.

## Supporting information


**Figure S1**: Purification of AcrVA3. SEC profile of purified AcrVA3, with eluted size markers indicated above the profile. The pictures of SDS–PAGE gel, loaded with the peak fractions are provided under the profile. Loaded fractions are indicated by black lines. M indicates protein marker.
**Figure S2**: Summary of the DALI search results.
**Figure S3**: Structure‐specific endonuclease activity test of AcrVA3.
**Figure S4**: The quantification results corresponding to Figure 1a,E,Fnd 2F. To provide a clearer quantification of target DNA cleavage by Cas12a and its inhibition by AcrIIVA3, we quantified both the substrate and product band intensities from the gel and expressed their ratio as the cleavage ratio. The quantification results corresponding to Figure 1E (A), Figure 1F (B), and Figure 2F (C) are presented. The experiments were performed independently three times and the mean values with standard deviations are presented. The numbers on the X‐axis correspond to the lane numbers shown below each gel in panels A–C.

## Data Availability

If derived from public domain information.
