# Supplementary material for: AcrVA3 Is a Double Strand DNA‐Cleaving Anti‐CRISPR That Indirectly Inhibits Cas12
Source: FASEB J. 2026 Mar 19;40(6):e71705. doi: 10.1096/fj.202502278RR (PMC13001698; doi:10.1096/fj.202502278RR)
Supplement: Supplementary file 1 — Figure S1: Purification of AcrVA3. SEC profile of purified AcrVA3, with eluted size markers indicated above the profile. The pictures of SDS–PAGE gel, loaded with the peak fractions are provided under the profile. Loaded fractions are indicated by black lines. M indicates protein marker. Figure S2: Summary of the DALI search results. Figure S3: Structure‐specific endonuclease activity test of AcrVA3. Figure S4: The quantification results corresponding to Figure 1a,E,Fnd 2F. To provide a clearer quantification of target DNA cleavage by Cas12a and its inhibition by AcrIIVA3, we quantified both the substrate and product band intensities from the gel and expressed their ratio as the cleavage ratio. The quantification results corresponding to Figure 1E (A), Figure 1F (B), and Figure 2F (C) are presented. The experiments were performed independently three times and the mean values with standard deviations are presented. The numbers on the X‐axis correspond to the lane numbers shown below each gel in panels A–C. [file FSB2-40-e71705-s001.pdf]

Supporting Information for

AcrVA3 is a double strand DNA-cleaving anti-CRISPR that indirectly inhibits Cas12

Ju Hee Han[1,2,#], Young Jun Kang[1,2,#], So Yeon Lee[1,2,#], Hyo Been Jin[1,2], Chang Sup Lee[3], and Hyun Ho Park[1,2,*]

[1]College of Pharmacy, Chung-Ang University, Seoul 06974, Republic of Korea
[2]Department of Global Innovative Drugs, Graduate School of Chung-Ang University, Seoul 06974, Republic of Korea
[3]College of Pharmacy and Research Institute of Pharmaceutical Science, Gyeongsang National University, Jinju 52828, Republic of Korea.

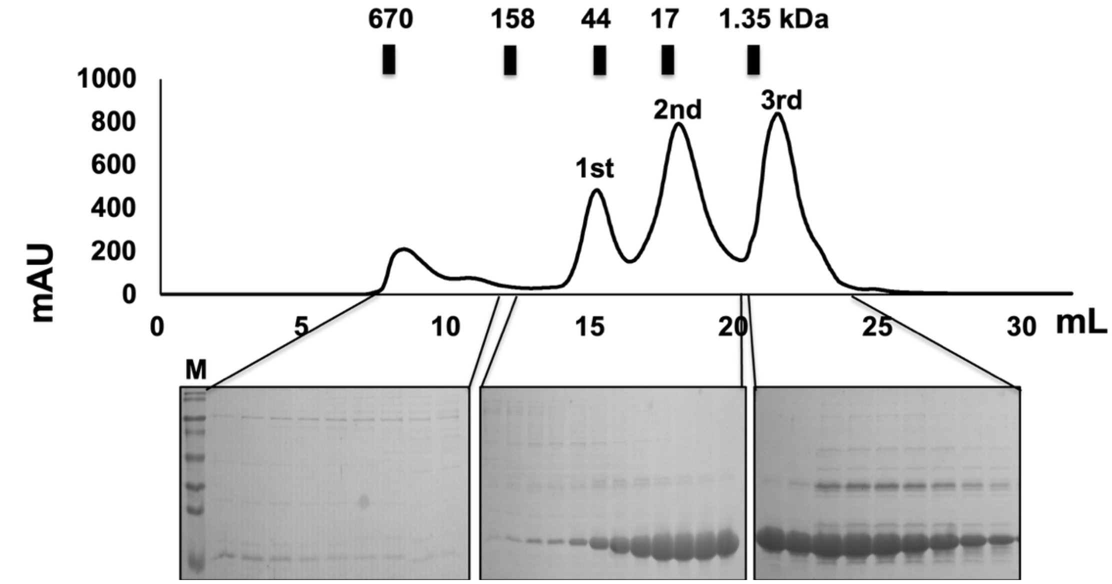

Supplementary Figure 1. Purification of AcrVA3. SEC profile of purified AcrVA3, with eluted size markers indicated above the profile. The pictures of SDS–PAGE gel, loaded with the peak fractions are provided under the profile. Loaded fractions are indicated by black lines. M indicates protein marker.

Proteins (accession numbers)	Z-score	RMSD (Å)	Identity (%)
Endonuclease I-HmuI (1U3E)	5.4	1.9	15
Protein kinase OSR1 (7OKW)	3.1	2.7	7
MCM-like helicase (5DGK)	3.1	3.0	15
WNK1 (5G3Q)	3.0	2.7	4
AcrIIA15 (8JFO)	2.6	3.1	12

Supplementary Figure 2. Summary of the DALI search results.

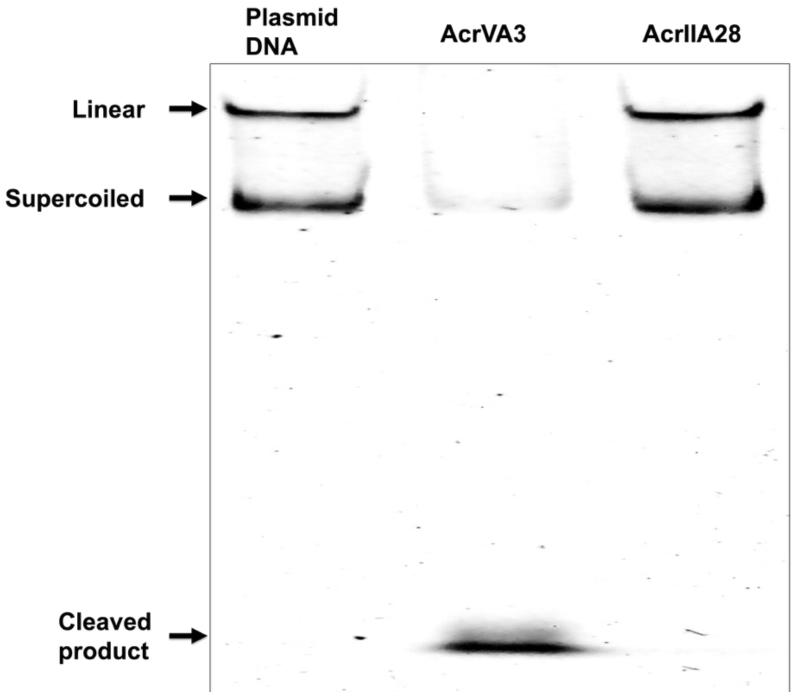

Supplementary Figure 3. Structure-specific endonuclease activity test of AcrVA3.

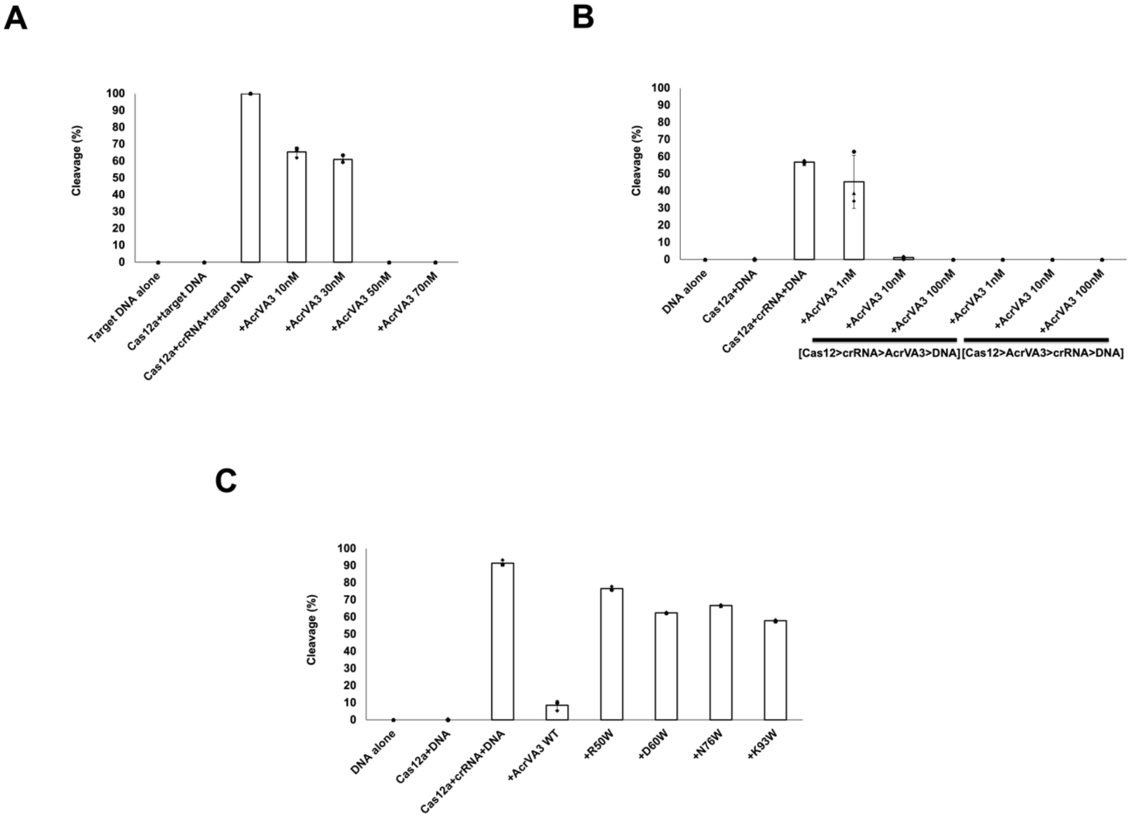

Supplementary Figure 4. The quantification results corresponding to Figure 1E, 1F, and 2F. To provide a clearer quantification of target DNA cleavage by Cas12a and its inhibition by AcrIIVA3, we quantified both the substrate and product band intensities from the gel and expressed their ratio as the cleavage ratio. The quantification results corresponding to figure 1E (A), figure 1F (B), and figure 2F (C) are presented. The experiments were performed independently three times and the mean values with standard deviations are presented. The numbers on the X-axis correspond to the lane numbers shown below each gel in panels A–C.
